# Annual Commencement of the Baltimore College of Dental Surgery

**Published:** 1851-04

**Authors:** 


*Annual Commencement of the Baltimore College of Dental Surgery.*—The twelfth annual commencement of this institution was held on Thursday, 27th March, at 11 o'clock, A.M., in the Assembly Room, corner of Hanover and Lombard streets. The exercises were opened with prayer by the Rev. Dr. Piggott; after which, Prof. T. E. Bond gave a brief history of the institution. Prof. Cone then made a report of the operations of the Infirmary, stating that 1700 cases had been successfully treated during the year. Prof. Handy stated the authority for conferring the degree of "Doctor of Dental Surgery," read the names of the candidates for the same, with the subjects of their theses; who were afterwards called up in classes of four, and the degree conferred on them by Dr. Harris. Dr. Eleazar Parmly, of New York, Provost of the Institution, then proceeded to deliver the Valedictory Address to the graduates, which was listened to with the most profound attention, not only by the class, but also by a very large and fashionable audience of ladies and gentlemen, who increased the interest of the occasion by their presence. The Address reflected great credit upon the distinguished author, abounded with much excellent advice to the graduates, and contained interesting biographies of the late Drs. L. Koecker, of London, and L. Roper, of Philadelphia. We hope to have an opportunity of presenting the Address entire to our readers in a future number of the Journal.

The exercises were interspersed with music from the Blues' Band.

The following are the names of the graduates: Rufus K. Chandler, of Va.; R. P. Bessant, of North Carolina; Wm. J. Reese, of Alabama; J. Randolph Walton, of Md.; George S. Jones, of Kentucky; Edward H. Howerton, of Va.; John A. Johns, M. D., of Va.. Edward S. Billups, of Georgia; Lloyd T. M'Gill, M. D., of Maryland; William S. Brown, of South Carolina; Thomas W. Bacot, do.; Richard M. Adair, of Kentucky; Ehrick Parmly, of New York; George S. Bretz, of Pa.; Thomas D. Miller, of England; James North, M. D., of Maine; Francis P. Abbot, do. Total, 17.

